# Absence of Endochondral Ossification and Craniosynostosis in Posterior Frontal Cranial Sutures of *Axin2^−/−^* Mice

**DOI:** 10.1371/journal.pone.0070240

**Published:** 2013-08-01

**Authors:** Björn Behr, Michael T. Longaker, Natalina Quarto

**Affiliations:** 1 Hagey Laboratory, Department of Surgery, Stanford University School of Medicine, Stanford, California, United States of America; 2 Department of Plastic and Hand Surgery, Burn Center, BG-University Hospital, Bergmannsheil, Ruhr-University Bochum, Bochum, Germany; 3 Dipartimento di Scienze Biomediche Avanzate, Universita’ degli Studi di Napoli Federico II, Napoli, Italy; Hospital for Sick Children, Canada

## Abstract

During the first month of life, the murine posterior-frontal suture (PF) of the cranial vault closes through endochondral ossification, while other sutures remain patent. These processes are tightly regulated by canonical Wnt signaling. Low levels of active canonical Wnt signaling enable endochondral ossification and therefore PF-suture closure, whereas constitutive activation of canonical Wnt causes PF-suture patency. We therefore sought to test this concept with a knockout mouse model. PF-sutures of *Axin2^−/−^* mice, which resemble a state of constantly activated canonical Wnt signaling, were investigated during the physiological time course of PF-suture closure and compared in detail with wild type littermates. Histological analysis revealed that the architecture in *Axin2^−/−^* PF-sutures was significantly altered in comparison to wild type. The distance between the endocranial layers was dramatically increased and suture closure was significantly delayed. Moreover, physiological endochondral ossification did not occur, rather an ectopic cartilage appeared between the endocranial and ectocranial bone layers at P7 which eventually involutes at P13. Quantitative PCR analysis showed the lack of *Col10α1* upregulation in *Axin2^−/−^* PF-suture. Immunohistochemistry and gene expression analysis also revealed high levels of type II collagen as compared to type I collagen and absence of Mmp-9 in the cartilage of *Axin2^−/−^* PF-suture. Moreover, TUNEL staining showed a high percentage of apoptotic chondrocytes in *Axin2^−/−^* PF-sutures at P9 and P11 as compared to wild type. These data indicated that *Axin2^−/−^* PF-sutures lack physiological endochondral ossification, contain ectopic cartilage and display delayed suture closure.

## Introduction

Mammalian skull vaults are composed of mesodermal and neural-crest derived bones and predominantly form through intramembranous ossification [1]. Bony growth occurs through differentiating mesenchymal cells at their edges, the so-called osteogenic fronts. When osteogenic fronts approximate each other, they can either fuse or form a cranial suture. Among the four main cranial sutures of the skull vault: paired coronal (COR), paired lamboid (LAM), sagittal (SAG) and posterior-frontal (PF) [2], the PF-suture is unique in the fact that it undergoes physiological closure [3], [4]. We have previously demonstrated that mouse PF-suture closure begins at P7 and ends between postnatal days 13 and 15, this process occurs through endochondral ossification [3]. An important regulator of skeletal development and endochondral ossification is canonical Wnt-signaling (cWnt). Wnt proteins bind to trans-membranous receptors Frizzled and Lrp 5/6. In the absence of active cWnt signaling, the central intracellular protein β-catenin is degraded by the destruction complex of dishevelled, adenomatous-polyposis-coli protein, glycogen synthase kinase-3β and axin [5], [6]. Upon activation of cWnt signaling, β-catenin is stabilized and translocates into the nucleus where it activates transcription factors such as TCF/LEF [5], [6]. With regards to endochondral ossification, the interplay between cWnt signaling and *Sox9*, the master gene for chondrogenesis is well described [7], [8]. Several studies have shown, that cWnt-signaling represses terminal chondrogenesis [9], [10], [11].

Recently, we have shown that endochondral ossification during PF-suture closure is tightly regulated by cWnt signaling [12]. By utilizing haploinsufficient *Axin2^+/−^* mice, the activation of cWnt signaling was found to be biphasic during suture closure. In the PF-suture mesenchyme, cWnt signaling was active until P7, followed by a decrease at P9 coinciding with cartilage formation. By the time chondrocytes underwent hypertrophy (P13), cWnt signaling was exclusively active in the chondrocytes and not detectable thereafter (>P15). Importantly, this pattern could be altered by exogenous application of Wnt3a protein on the PF suture. Mice continuously treated with Wnt3a exhibited PF suture patency [12]. In an additional study, we could show that coronal craniosynostosis in *Twist1^+/−^* mice occurred through endochondral ossification [13]. Moreover, we compared the activity of cWnt signaling between the four different calvarial sutures, which suggested a strict correlation between high cWnt activity and suture patency [13].

A genetical model to study increased cWnt-signaling is the *Axin2-lacZ^−/−^* reporter mouse [12], [15]. *Axin2* is a negative regulator of the cWnt pathway and has several Tcf/LEF consensus binding sites in the promoter/first intron [14]. In conjunction with glycogen synthase-3β and adenomatosis polyposis protein, *Axin2* promotes the ubiquitination and degradation of β-catenin, leading to inhibition of cWnt-signaling [14]. It has been previously reported, that in *Axin2^−/−^* mice the PF-suture fuses prematurely at P8 [15]. The authors concluded, that *Axin2^−/−^* mice resemble a phenotype equivalent to craniosynostosis in humans [15].

Given our recent studies and compelling evidence from the literature, the apparent contradiction that increased cWnt signaling as present in *Axin2^−/−^* mice results in premature PF-suture closure had to be investigated. Therefore, we reasoned to revisit the PF-suture of *Axin2^−/−^* mice in detail and study its morphology and development during the physiological time frame of its closure.

## Materials and Methods

### Animals

All experiments using animals were performed in accordance with Stanford University Animal Care and Use Committee guidelines (protocol ID #APLAC 8397). The Institutional Animal Care and Use Committee (IACUC) specifically approved this study. *Axin2-lacZ* ^−/−^ homozygotic mice were on a CD1 background and genotyped as previously described [15]. For all experiments, *Axin2-lacZ* ^−/−^ homozygotic mice and CD1 wild type littermates were used.

### Tissues Harvesting and Processing

Animals (n = 7) for each time point were sacrificed on the exact postnatal day (P) based on birth date. PF-sutures were harvested, dissected under a stereomicroscope (Stemi 2000 Zeiss, Thornwood, NY), as previously described [3], and processed individually. Specimen were fixed in 4% PFA at 4°C overnight and then decalcified with 19% EDTA for the appropriate time. Thereafter, specimens were dehydrated through a series of alcohol and paraffin embedded. For cryo-sections, specimen were fixed in 0.4% PFA, decalcified in 19% EDTA, transferred to 30% sucrose and embedded in optimal cutting temperature (OCT). For qPCR analysis, PF-sutures from mice at different time points were harvested as previously described [3]. The ectopic cartilage was meticulously dissected from the PF-sutures under a StereoMicroscope (Stemi 2000, Zeiss, Thornwood, NY) and processed for gene expression analysis.

### RNA Isolation and Reverse Transcription-PCR Analysis

Procedures for RNA isolation, reverse transcription and quantitative PCR (qPCR) were previously described [3], [16]. Briefly, qPCR was performed using the ABI Prism 7900 Sequence Detection System, TaqMan Gene Expression Master Mix, and TaqMan Gene Expression Assays (Applied Biosystems, Foster City, CA). qPCR reactions were performed under the following conditions: 94°C for 5 minutes, 94°C for 30 seconds, annealing at 60°C for 1 minute and 72°C for 1 minute (25–30 cycles). *Gapdh, Sox9, Col2α1, Col1α1, Col10α1 and Bglap* primers have been previously described [3], [16]. *Mmp-9* primers sequence is as follows:

*Mmp-9* (Forward), 5′-GGAACTCACACGACATCTTCCA-3′;

*Mmp-9* (Reverse), 5′-GAAACTCACACGCCAGAAGAATTT-3′.

All results are presented as the mean ±SD of three independent experiments.

### Histology and *in situ* TUNEL Assay

Entire PF-sutures were cut in 10 µm sections. For paraffin-sections, every sixth slide was stained with pentachrome to determine the exact region within the suture. For TUNEL staining of DNA-strand breaks, sections were incubated with Proteinase K (Roche, Indianapolis, IN) for 10 minutes followed by TUNEL reaction mix (*In situ* cell death detection kit, Roche). Sections were mounted with Nuclear counterstaining was performed on all cells using Vectashield H-1200 mounting medium with DAPI (Vector Laboratories, Burlingame, CA) and evaluated under an epifluorescene microscope (Leica DFC 500). Cryo-sectioned slides were stained with X-Gal (Roche, Indianapolis, IN). Sections were examined with a Carl Zeiss Axioplan 2 (Zeiss, Thornwood, NY) microscope. Images were captured by AxioVision software (Zeiss). Apoptotic and total cell numbers were manually counted within the cartilage area by two independent examiners. Histomorphometry results represent the mean of three TUNEL-stained PF-suture sections, containing cartilage (n = 3 of each time point).

### Immunohistochemistry

Antigen retrieval was performed as previously described [12]. Antibodies against type I and II Collagen (Santa Cruz Biotechnology, Santa Cruz, CA) were used in a dilution 1∶50, Mmp-9 (R&D Systems, Minneapolis, MN) in a dilution of 1∶100. A goat or rabbit biotinylated secondary antibody followed by the AB reagent and NovaRed (Vector Laboratories, Burlingame, CA) were used for detection according to manufacture’s instructions. Goat or Rabbit irrelevant IgG (Calbiochem, Rockland, MA) used as a negative control, did not detect any staining (data not shown).

### Animal Surgery

Skin incision was performed above the PF suture of anesthetized P4 mice, n = 7 for each treatment group. A 1.5 mm diameter collagen sponge (Helistat, Integra LifeSciences, Plainsboro, NJ) was soaked with a combination of 2 µg of secreted Frizzled related protein-1 (sFrp1) and 2 µg Dickkopf-1 (Dkk1) (R&D Systems, Minneapolis, MN) and placed on the PF-suture, These two inhibitors were applied in combination to ensure a proper inhibition of the LRP5/6 and Frizzled receptor, as well as sequestration of Wnt ligands in the extracellular space as previously described [12]. PBS-soaked collagen sponges served as controls. The skin incision was then closed and mice were allowed to recover. Every other day, the skin incision was reopened, the sponge was re-soaked with the corresponding factor(s) or PBS. Then, the skin was closed. Sham operated animals (n = 5) were also included into the experiment, giving results similar as PBS-soaked collagen controls (data not shown).

### Morphometric Analysis

Distances between the endo- and ectocranial bone layers of the PF-suture were measured with the ruler tool in the AxioVision software (Zeiss, Thornwood, NY) in triplicate for each time point. Distances between the endo- and ectocranial layers were defined as the minimum length between the two osteogenic fronts. The selection of X-gal positive pixels was partially automated with the magic wand tool (tolerance: 60, no contiguous) in Photoshop (Adobe, San Jose, CA). In order to determine the cartilage surface, the elliptical marquee tool was utilized. All images were cropped with a rectangular that covered the entire suture (4×10^6^ pixels). The number of positive pixels was recorded and an average for each tissue sample was generated. Thereafter, averages for each group were calculated.

### Statistical Analysis

Data are expressed as mean ± SD of at least three independent samples. Statistical comparisons between groups were performed with a two-tailed Student’s t-test, *P≤0.05 and **P≤0.01 was considered significant.

## Results

### Endochondral Ossification and Suture Closure is Impaired in PF-suture of *Axin2^−/−^* Mice

**Figure 1:**
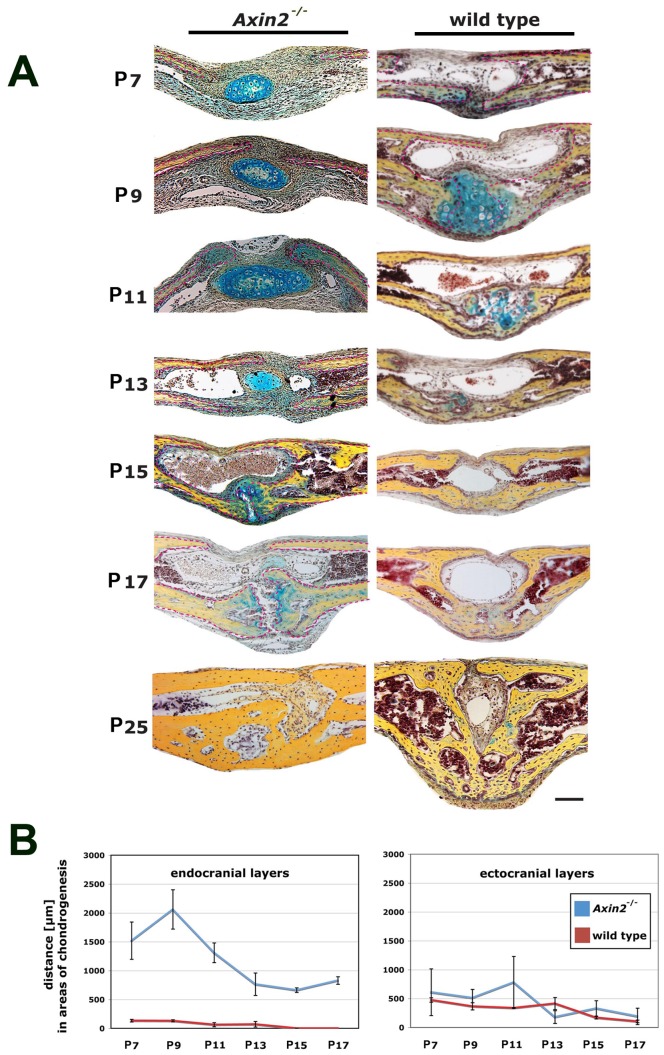
Lack of endochondral ossification and closure delay in *Axin2^−/−^* PF-sutures. **A**, pentachrome staining of PF-sutures from *Axin2^−/−^* and wild type mice during the timing of physiological closure reveals an involuting ectopic cartilage in the suture mesenchyme of *Axin2^−/−^* PF-sutures, while endochondral ossification occurs in wild type PF-sutures. Closure is delayed in *Axin2^−/−^* PF-sutures compared to wild type PF-sutures. *Axin2^−/−^* PF-sutures closure is observed at day P25. **B,** measurements of the distance between the approaching osteogenic fronts of endocranial and ectocranial bone plates in *Axin2^−/−^* and wild type PF-sutures show significantly greater distances between the osteogenic fronts of the endocranial bone plates in *Axin2^−/−^* PF-sutures from P7 to P17. Scale bar: 100 µm.

*Axin2^−/−^* mice have a constitutively active cWnt signaling due to a lack of the internal inhibitor *Axin2*, which is part of the GSK3β-degradation complex [5], [6]. To investigate in detail potential effects of constant cWnt signaling activation on the PF-sutures of *Axin2^−/−^* mice, we compared serial coronal sections of entire PF-sutures harvested from *Axin2^−/−^* and CD-1 wild type mice side-by-side. Striking differences in the morphology of the *Axin2^−/−^* and wild type PF-suture were observed (**Figure 1A**). At postnatal day 7 (P7), pentachrome staining revealed a bulk of premature and ectopic cartilage within an expanded suture mesenchyme, though this cartilage was not in contact with the osteogenic fronts. Moreover, the endocranial bone layers were dramatically less developed, whereas in wild type mice, these bone layers already approximated each other with the onset of endochondral ossification, as observed previously [3]. At P9, this ectopic cartilage persisted caudal of the ectocranial bone layers of *Axin2^−/−^* mice while, in wild type PF-sutures cartilage undergoing endochondral ossification formed between the endocranial layers. The first bony bridge between the osteogenic fronts in wild type PF-sutures was observed at P11, in contrast, the endocranial bone layers were still underdeveloped in *Axin2^−/−^* PF-sutures and osteogenic fronts were far apart. By day P15, endochondral ossification and PF-sutures closure were completed in wild type mice. Conversely, PF-sutures of *Axin2^−/−^* mice were still open and in the medial part of the approximating endocranial layers a faint staining for cartilage was observed. A similar pattern was observed at day P17. By day P25, *Axin2^−/−^* PF-sutures were fused but showed decreased height and robustness as compared to wild type mice. Thus, as previously described in wild type mice [12], PF-suture fusion started with endochondral ossification at day p9, formed a bony bridge at day P11 and completed by day P15. In sharp contrast, PF-suture closure in *Axin2^−/−^* mice was delayed without a clear pattern of endochondral ossification as observed in wild type mice. Interestingly, we observed cartilage in the suture mesenchyme without contact to the endocranial or ectocranial layers, and this ectopic cartilage seemed to involute over time.

We next compared the distance between the osteogenic fronts of the endocranial and ectocranial bone layers, both in *Axin2^−/−^* and wild type mice (**Figure 1B**). The distance between endocranial bone layers in *Axin2^−/−^* PF-sutures markedly widened during development. At P7 the mean distance was 1518 µm in *Axin2^−/−^* as compared to 132 µm in wild type mice. After a peak by day P9, the distance between the osteogenic fronts of the endocranial bone layers in *Axin2^−/−^* and wild type mice decreased during the following time points, however at P17 it was still significantly higher (828 µm) in *Axin2^−/−^*, whereas in wild type mice the endocranial layers were fused. For the ectocranial layer, the distance between the osteogenic fronts was narrower in *Axin2^−/−^* as compared to wild type mice. At P7, the distance was 608 µm in *Axin2^−/−^* and 473 µm in wild type mice. Over the time course, the distance of the ectocranial layers in wild type mice likewise decreased.

### Cartilage of *Axin^−/−^* PF-sutures does not Undergo Hypertrophy

**Figure 2:**
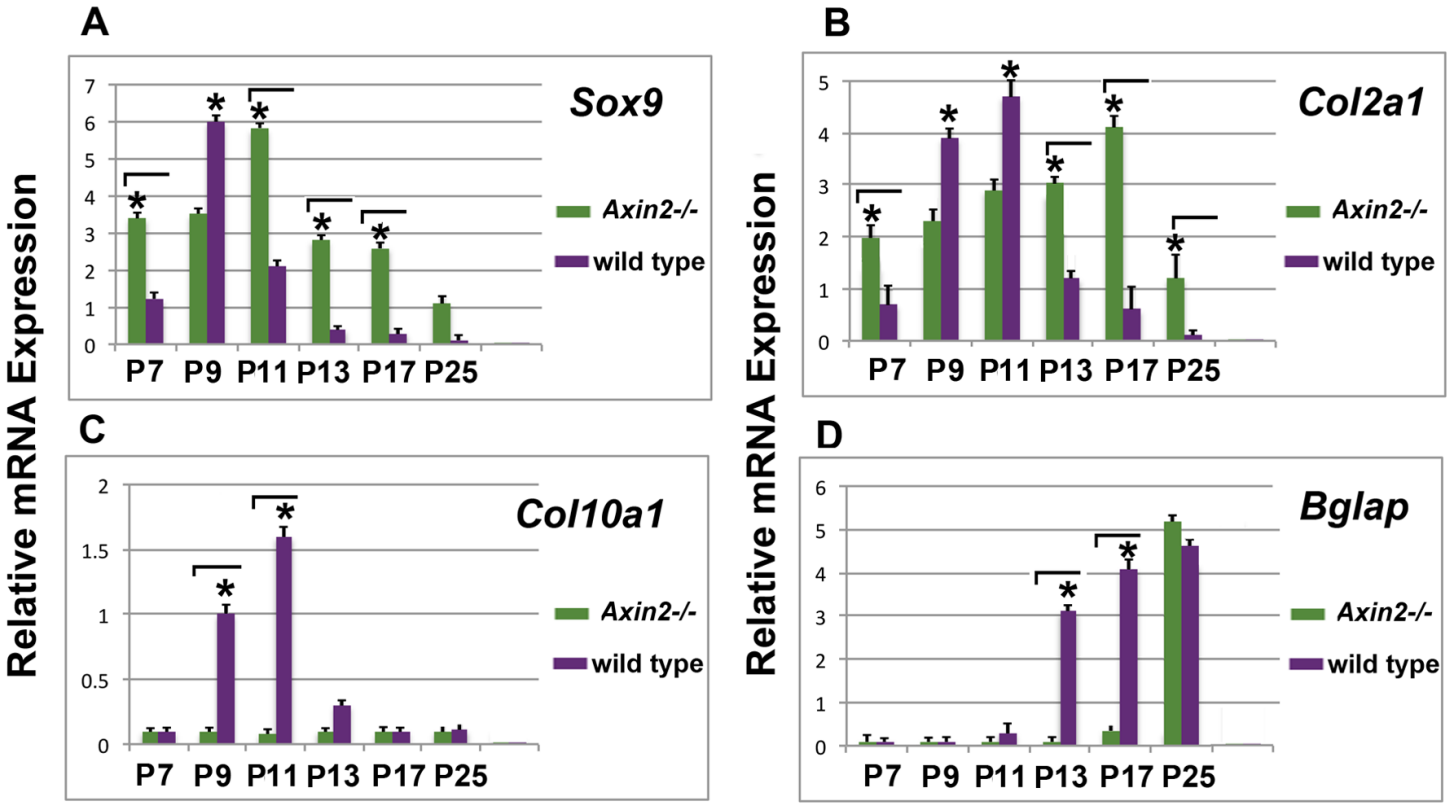
Chondrogenic profile of *Axin2^−/−^* and wild type sutures. **A,** qPCR analysis of chondrogenic markers at different time points coinciding with timing of physiological closure of PF-sutures through endochondral ossification. Expression profile of cartilage signatures gene is unique in *Axin2^−/−^* PF-sutures compared to wild type PF-sutures. Elevated levels of *Sox9* gene expression are observed starting from day P7 and remain sustained at later time points. Expression of *Col2α1* also is higher (P13 and P17) and sustained compared to wild type PF-sutures. In contrast to wild type PF-sutures, in *Axin2^−/−^* PF-sutures is not detected upregulation of *Col10α1* gene, a marker of hypertrophic chondrocytes. By day P13, expression of the osteocalcin gene *Bglap* is already upregulated in wild type PF-sutures. Conversely, in *Axin2^−/−^* PF-sutures *Bglap* expression is observed at P25. The relative mRNA level in each sample is normalized to its *Gapdh* content. Values are given as relative to *Gapdh* expression.

Thus, in *Axin2^−/−^*mice pentachrome staining revealed an unusual pattern of PF-sutures closure characterized by the presence of ectopic involuting cartilage and delayed timing of closure. Therefore, we analyzed *Axin2^−/−^* PF-suture closure at a molecular level. As shown in **Figure 2**, qPCR analysis of signature genes of chondrogenesis, *Sox9, Col2α1* and *Col10α1* revealed qualitative as well as quantitative differences in the expression profile between *Axin2^−/−^* and wild type PF-sutures. Wild type PF-sutures showed a temporal upregulation of *Sox9*, *Col2α1* and *Col10α1* (**Figure 2** **A–C**) at specific time points, as previously described, during the timing of PF-sutures closure which is known to occur through endochondral ossification [3]. By P13, when the process of PF-suture closure was already completed in wild type mice, we observed upregulation of the osteocalcin gene *Bglap*, and the presence of newly formed bony tissue (**Figure 2D**). In contrast, a unique gene expression profile was observed in *Axin2^−/−^* PF-sutures. At day P7, *Sox9* expression in *Axin2^−/−^* PF-sutures was already higher when compared to wild type PF-sutures, where its upregulation occurred by day P9. Expression of *Col2α1* gene was overall significantly higher (P13-P17) and remained sustained until day P17 in *Axin2^−/−^* PF-sutures. Interestingly, upregulation of *Col10α1* was not observed as in wild type PF-sutures. By day P25, upon suture closure, *Bglap* was unregulated in *Axin2^−/−^* PF-suture as a result of bony tissue formation. However, upregulation of this gene could be detected much earlier in wild type PF-sutures at day P13. The gene expression analysis above, reveals absence of hypertrophic cartilage in *Axin2^−/−^* PF-sutures, and therefore suggests that closure of this suture does not occur through endochondral ossification.

### Cartilage in *Axin2^−/−^* PF-suture is not a Template for Endochondral Ossification

**Figure 3:**
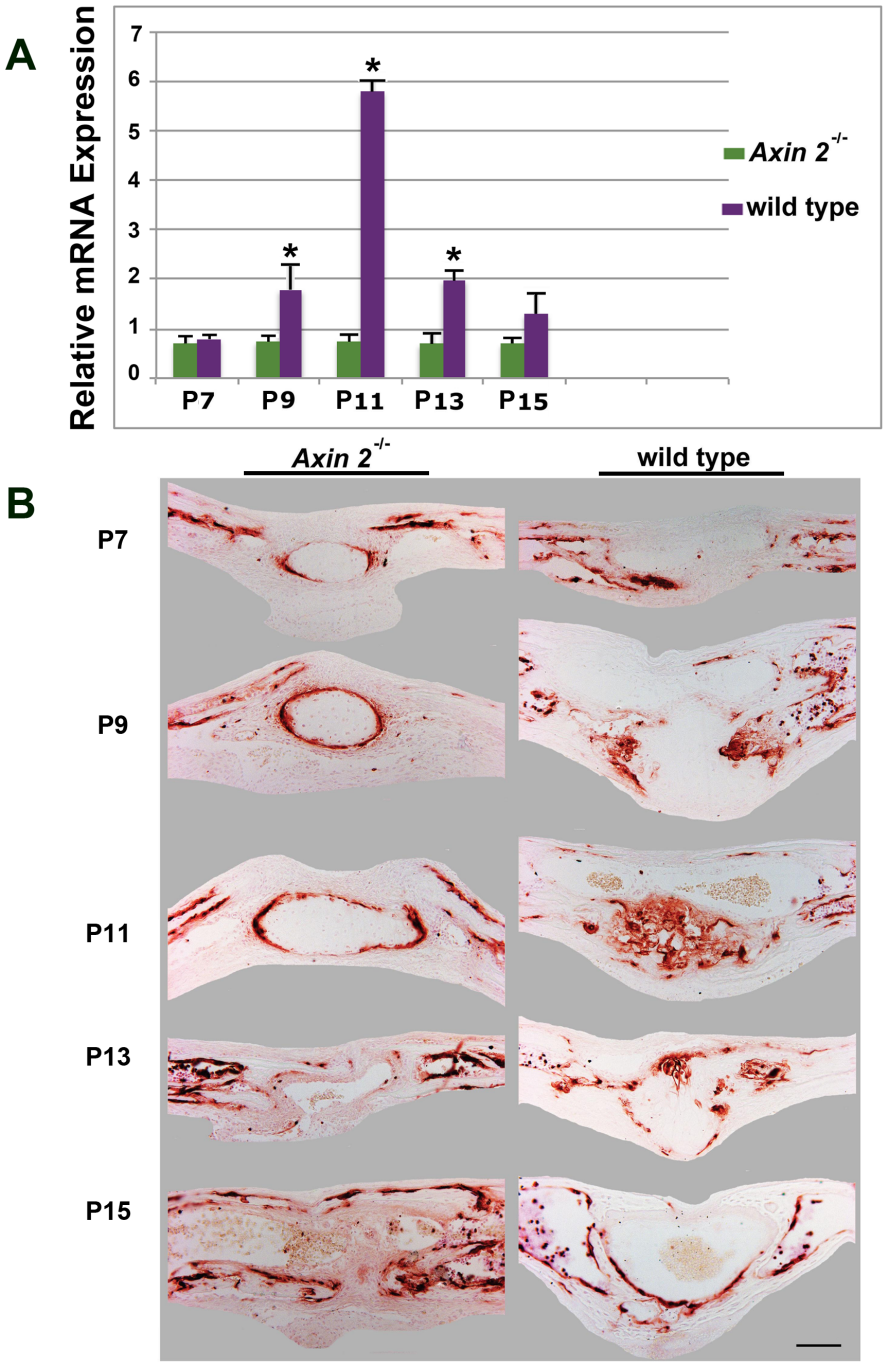
Comparative analysis of Mmp-9 between *Axin2^−/−^* and wild type sutures. **A,** **** *Mmp-9* gene expression analysis by qPCR at different time points reveals extremely low levels of *Mmp-9* expression in *Axin2^−/−^* PF-sutures. In contrast, in wild type PF-sutures is observed upregulation of *Mmp-9* starting by day P9 with a peak at day 11. **B,** MMP-9 immunohistochemistry does not detect protein in the cartilage area of *Axin2^−/−^* PF-sutures, whereas strong staining is detected in the hypertrophic cartilage of wild type PF-suture at day P11. Scale bar: 100 µm.

Both, pentachrome staining and gene expression profile analysis indicated that the ectopic cartilage within the *Axin2^−/−^* PF-sutures, did not achieve the hypertrophy stage and involuted rather than progressing to endochondral ossification. To further confirm this observation, we analyzed the expression profile of *Mmp-9*, a key regulator of hypertrophic cartilage degradation and vascularization [17] expressed by chondroclasts [18]. Time course qPCR analysis of *Mmp-9* gene expression showed downregulation of this gene in *Axin2^−/−^* PF-sutures. In contrast, a distinct expression profile was observed in wild type PF-sutures characterized by *Mmp-9* upregulation at day P11, followed by a marked decreased at day P13 (**Figure 3A**). Similarly, Mmp-9 protein was not detected in the cartilage area of *Axin2^−/−^* PF-sutures and Mmp-9 staining was observed only outside the cartilage area as shown by immunohistochemistry (**Figure 3B**). Conversely, intense Mmp-9 staining was observed in the cartilage of wild type PF-sutures at day P11 (**Figure 3B**). These results further suggest that indeed, the cartilage present in the *Axin2^−/−^* PF-sutures did not progress through hypertrophy.

**Figure 4:**
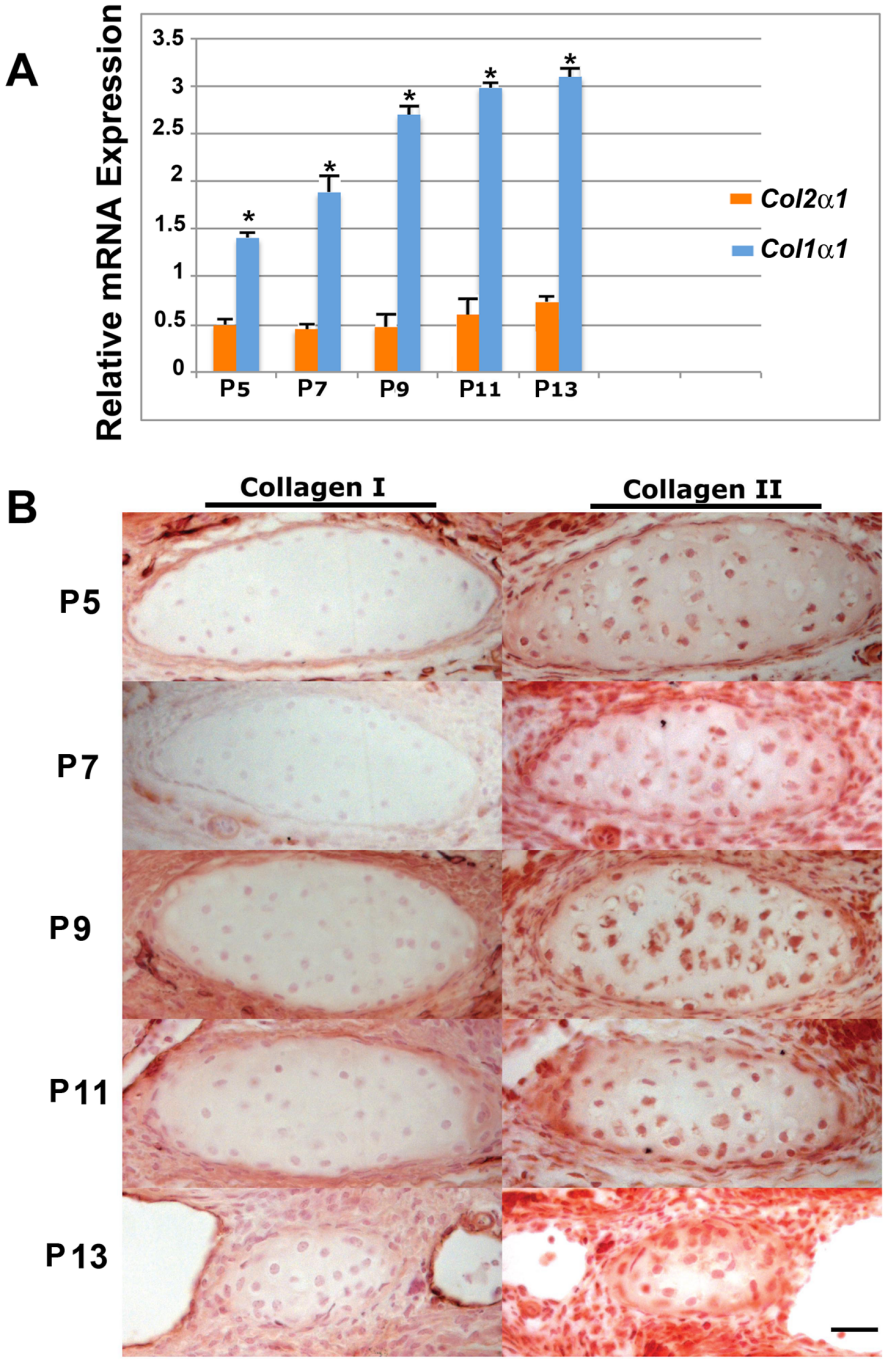
Characterization of ectopic cartilage in *Axin2^−/−^* suture. **A**, comparative qPCR analysis of *Col2α1* and *Col1α1* genes expression in *Axin2^−/−^* PF**-**sutures shows overtime elevated levels of *Col2α1*, whereas *Col1α1* expression is significantly lower. **B,** immunohistochemistry of type I and type II collagen performed on coronal sections of *Axin2^−/−^* PF-sutures. Chondrocyte staining is more intense for type II Collagen as compared to type I Collagen, indicating a hyaline nature of the cartilage Scale bar: 50 µm.

As a next step, we sought to identify the nature of this cartilage in more detail. Specifically, we were interested to know whether the ectopic cartilage in *Axin2^−/−^* PF-sutures could be of hyaline nature. To verify this possibility we performed *Col2α1* and *Col1α1* gene expression analysis (**Figure 4A**) and immunohistochemistry for type Collagen I and Collagen II proteins (**Figure 4B**), as it is well established that the hyaline cartilage is high in Collagen type II but very low in Collagen type I content. In a time course analysis performed on *Axin2^−/−^* PF-sutures, at both gene and protein level, we found higher levels of type II Collagen as opposed to Collagen I, overtime (**Figure 4A and 4B** **)**. This profile was different from the spatio-temporal profile usually observed in wild type PF-sutures undergoing endochondral ossification and closure [3]. It is well characterized indeed, that wild type PF-sutures display a distinct expression pattern of these two collagen genes, with *Col2α1* upregulated at day P9 and P10 followed by downregulation starting from day P11, while *Col1α1* expression has a biphasic upregulation, first from P6 to P9 and second from day P11 [3]. Taken together, the above analyses strongly suggest that the ectopic cartilage found in *Axin2^−/−^* PF-suture is of hyaline nature.

### *Axin2^−/−^* PF-suture Chondrocytes Undergo Apoptosis

**Figure 5:**
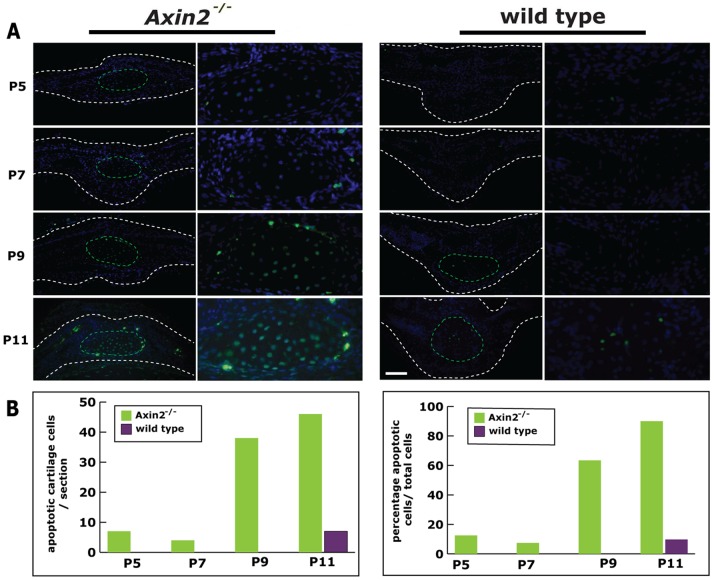
Apoptotic activity in *Axin2^−/−^* and wild type sutures. **A,** **** TUNEL staining performed from day P5 to P11 reveals increased apoptosis in chondrocytes of *Axin2^−/−^* PF-sutures as opposed to wild type PF-sutures at P9 and P11. **B,** Quantification of apoptosis shows significantly more apoptotic chondrocytes per/section (**left panel**) and higher percentage of apoptotic chondrocytes in relation to the total cell number (**right panel**) in *Axin2^−/−^* PF-sutures as compared to wild type PF-sutures. Scale bar: 100 µm.

Pentachrome staining revealed in *Axin2^−/−^* PF-suture the presence of ectopic cartilage which does not interact with the osteogenic fronts of the suture and involutes between day P13 and P15 (**Figure 1**). In order to test, whether this process may be related to apoptosis, we performed an *in situ* TUNEL staining (**Figure 5A and 5B**). TUNEL staining revealed more apoptotic chondrocytes in *Axin2^−/−^* PF-sutures compared to wild type suture **(** **Figure 5A** **).** Quantification showed overtime a dramatic increase in apoptosis of cartilage cells at day P9 (mean: 38 cells/section) and P11 (mean: 45 cells/section) in *Axin2^−/−^* PF-sutures as compared to wild type, where cells underwent apoptosis only during the stage of hypertrophic cartilage P11 (mean: 7 cells/section) as expected during endochondral ossification **(** **Figure 5B** **)**. The total ratio of cartilage cells undergoing apoptosis was up to 90% at p11 in *Axin2^−/−^* PF-suture, while it was 9.8% in wild type PF-suture **(** **Figure 5B** **)**.

### Partial Inhibition of cWnt-signaling Decreases Cartilage Size

**Figure 6:**
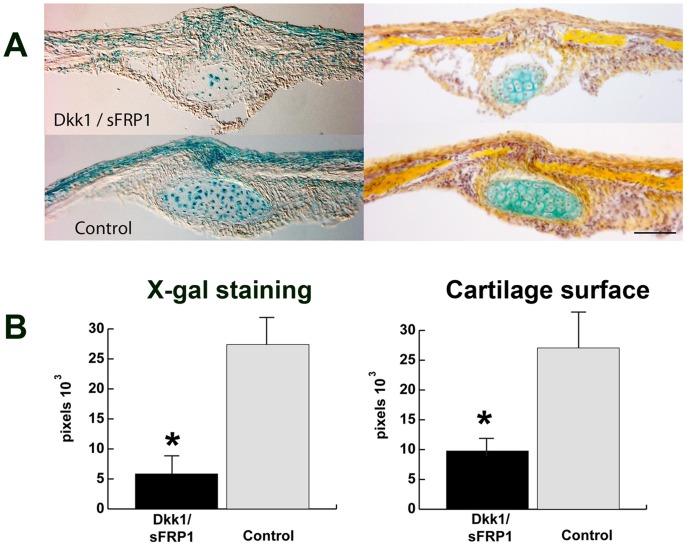
Partial inhibition of cWnt-signaling decreases ectopic cartilage size. **A,** X-gal staining, representative of endogenous active cWnt signaling, performed on P8 *Axin2^−/−^* PF-suture treated with the inhibitors of cWnt signaling Dkk1 (2 µg) and sFRP1 (2 µg) (n = 7) and untreated control (n = 7). X-gal staining is reduced in both cartilage areas as well as in the ectocranial layer and pericranium upon treatment with inhibitors (**left panel**). Pentachrome staining of adjacent slides reveals diminished cartilage in *Axin2^−/−^* PF-suture treated with Dkk1 and sFRP1 (**right panel**). **B**, Histomorphometry of X-gal staining and cartilage surface confirmed these findings. Quantification of X-gal staining (**left panel**) showing inhibition of cWnt signaling (73.8%) in Dkk1 and sFRP1 treated PF-suture compared to untreated control. In PF-suture treated with inhibitors the cartilage size is reduced by (63.8%) compared to untreated control (**right panel**). Scale bar: 100 µm.

In order to test, whether inhibition of cWnt signaling would alter PF-suture morphology and decrease the ectopic cartilage, *Axin2^−/−^* PF-sutures (n = 7) were continuously treated with inhibitors of cWnt signaling Dkk1 and sFRP1 (**Figure 6A**). X-gal staining, which monitors the degree of endogenous activation of cWnt signaling in this transgenic mice [15], was more intense in untreated *Axin2^−/−^* PF-suture controls (n = 7) as compared to suture treated with inhibitors. In particular, X-gal staining was more prominent in the cartilage of untreated *Axin2^−/−^* PF-sutures as well as in the suture mesenchyme between and cranial of the endocranial layers (**Figure 6A**). However, this treatment inhibited cWnt signaling only partially by 73.8%, similarly as previously observed in *Axin2^+/−^* SAG-suture [12]. The lack of complete inhibition of cWnt signaling by Dkk1 and sFRP1 could represent a potential explanation why this treatment was not sufficient to accelerate *Axin2^−/−^* PF-sutures closure by P16 (data not shown). In this experimental context, we cannot rule out that our surgery procedure could stimulate Wnt signaling, and counteract in part, the effect of inhibitors. However, activation of Wnt signaling would likely occur at skin level.

Although, inhibition of Wnt signaling was not complete, pentachrome staining of adjacent slides revealed a smaller cartilage area in *Axin2^−/−^* PF-suture treated with inhibitors as opposed to untreated control (**Figure 6A**), thus suggesting that partial inhibition of cWnt signaling was sufficient to decrease significantly the size of ectopic cartilage in *Axin2^−/−^* PF-suture. Quantification of cartilage area in *Axin2^−/−^* PF-suture treated with specific inhibitors of cWnt signaling indicated a reduction of ectopic cartilage area by 63.8% compared to untreated control (**Figure 6B**).

## Discussion

We have previously demonstrated that differential activation of cWnt signaling determines cranial sutures closure. The current study focuses on constitutive activation of cWnt signaling and represents a detailed comparative analysis of *Axin2^−/−^* and wild type PF-sutures. This analysis reveals that PF-sutures of *Axin2^−/−^* mice did not undergo physiological endochondral ossification, contained ectopic cartilage and displayed delayed suture closure. These data are consistent with previous findings, showing that constant activation of cWnt signaling resulted in inhibition of endochondral ossification [11], [12], [19]. Interestingly, we did observe ectopic cartilage, which did not undergo endochondral ossification but involuted presumably through apoptotic events. It is tempting to speculate that the presence of this ectopic cartilage, as a bulk, might induce compressive forces, which could delay the bony edges of endocranial layers, surrounding the PF-suture, from bridging. This hypothesis is intriguing and deserves further investigation.

It is well profiled that during endochondral ossification, *Sox9* is highly expressed in chondrocytes of the proliferating and prehypertrophic zone but downregulated abruptly in the hypertrophic zone, suggesting that *Sox9* downregulation is necessary step to initiate cartilage-bone transition [20]. Our gene expression analysis on *Axin2^−/−^* PF-sutures revealed sustained levels of *Sox9* expression throughout the entire time of cartilage presence in the PF-sutures, as well as lack of *Col10α1* a marker of hypertrophic chondrocytes. It is known that the transcription factor Sox9 activates *Col2α1* expression [21] therefore, this would explain the *Col2α1* expression profile we observed in *Axin2^−/−^* PF-sutures. Analysis by qPCR detected continuously higher expression levels of *Col2α1*, whereas *Col1α1* expression was very low. Taken together, these findings suggest firstly, that the cartilage observed in *Axin2^−/−^* PF-sutures does not undergo to hypertrophy, and secondly, that this cartilage is hyaline cartilage. Therefore, this ectopic cartilage does not serve as a template for the endochondral ossification of PF-suture closing. The cartilage detected in PF-sutures of *Axin2^−/−^* mice has characteristics similar to the cartilage observed in the developing mouse parietal and interparietal bones [22]. Here, a gradual disappearance of the cartilage was described until day P10. Interestingly, this cartilage removal did not occur by endochondral ossification, since the authors likewise found expression of *Col2α1* but not *Col10α1* and demonstrated apoptosis of chondrocytes by TUNEL staining [22]. The timing, as well as, the involution of cartilage in the posterior skull described in the aforementioned study may therefore be similar to the ectopic cartilage we observed in PF-sutures of *Axin2^−/−^* mice.

Noteworthy, in our previous study we also consistently observed physiologically ectopic cartilage caudal of SAG-sutures, which disappeared after day P7 [12]. It must be pointed out that the SAG-suture is characterized by constitutive activation of endogenous cWnt signaling [12], therefore it is tempting to speculate that high activation of cWnt signaling might serve as a permissive condition for the development of such an ectopic involuting cartilage as seen in *Axin2^−/−^* PF-sutures. Interestingly, the presence of an intermediate cartilage caudal of forming cranial suture seems to be conserved among biological classes, as recently reported in the amphibian model, Xenopus laevis [23]. In coronal sections derived from Xenopus frontoparietal bone, an involuting cartilage was detected at tadpole stage 56, shortly before the bone plate fused through intramembranous process [23].

Timely apoptosis of hypertrophic chondrocytes during endochondral bone formation is an integral and essential part of the endochondral ossification program [17]. However, our data in agreement with a previous study by Holmbeck et al. [22] demonstrate that apoptosis of non-hypertrophic chondrocytes is necessary also for the involution of ectopic cartilage in *Axin2^−/−^* PF-sutures. In wild type PF-sutures apoptosis was coupled with the hypertrophic stage of chondrocytes and high levels of the matrix metalloproteinase Mmp9. This resembled endochondral ossification, and was observed by day P11. In contrast, in *Axin2^−/−^* PF-sutures, apoptotic events started as early as day P5 and was sustained until P11. Furthermore, apoptosis was not mirrored by elevated levels of Mmp9. These data are in agreement with a previous study showing increase in apoptotic cells and deficiency in *Mmp9* expression in the area of non-hypertrophic cartilage of transgenic mice harboring *Sox9* misexpression [20].

Notably, the current data obtained from *Axin2^−/−^* homozygotic mice represent an exquisite example of a “gene-dosage effect”, since half-dose of the gene utilizing *Axin2^+/−^* haploinsufficient mice is not sufficient to alter PF-sutures anatomy and closure [12]. In addition, mice haploinsufficient for GSK-3β, an additional molecule of the intracellular β-catenin destruction complex [5], [6], likewise displayed no abnormal PF-suture morphology and closure (data not shown). Unfortunately, owing.

to embryonic lethality, the GSK-3β ^−/−^ mice are not available for analysis of PF-sutures.

Upon ectopic cartilage disappearance, the PF-suture eventually closed by P25. Given the absence of *Col10α1* gene expression and no histological evidence for endochondral ossification, but rather for hyaline cartilage formation, we can confidently conclude that PF-suture closure in *Axin2^−/−^* is a process occurring through intramembranous ossification.

The current work reveals results different from findings of a previous study, where the authors found premature fusion of PF-suture occurring at P8 in *Axin2^−/−^* mice [15] Of note, in their study PF-suture is referred to as metopic suture, a nomenclature used for the human PF-suture only [2]. There are several aspects, which could explain the gross discrepancy between the two studies: first, a discrepancy in the nomenclature and anatomy as well. The previous study was performed on the “metopic suture” (PF-suture), which was referred to by the authors as a suture lying between the nasal bones [15]. The PF-suture, however, lies between the frontal bones [3], [4]. Moreover, it is anatomically established, that the jugulum limitans, which marks the anterior border of PF-suture, divides anterior and posterior frontal bones [3], [4] and not frontal and nasal bones as stated [15]. Moreover, Yu and colleagues showed a low magnification anatomy of fusing *Axin2^−/−^* PF-sutures at P8. Second, Yu and colleagues did not analyze endochondral ossification or cartilage in *Axin2^−/−^* or wild type PF-sutures, although physiological PF-suture closure occurs through endochondral ossification between day P7 and P13 as previously reported [3]. Moreover, based on these data, P8 should not be considered a time point for premature suture closure. Third, it has to be stressed, that the potential relevance of a different genetic background strain cannot be ignored, considering significantly different baseline bone phenotypes among strains [24]. While the genetic background in our study was CD1, Yu et al. used a mixed 129 and C57Bl6, which limits the comparison to our study to some degree.

In summary, these data confirm the detrimental effects of constitutive activation of cWnt signaling on endochondral ossification in physiological PF-sutures closure, and challenge a previous work, which supposed that *Axin2^−/−^* PF-sutures display premature suture fusion. Our results showed that *Axin2^−/−^* PF-sutures have dramatically altered morphology, containing ectopic cartilage, and display a delay in closure. We therefore, have to conclude, that absence of *Axin2* does not cause PF-suture craniosynostosis.

## References

[1] Jiang X, Iseki S, Maxson RE, Sucov HM, Morriss-Kay GM (2002) Tissue origins and interactions in the mammalian skull vault. Dev Biol 241: 106–116. 11784098 10.1006/dbio.2001.0487

[2] Opperman LA (2000) Cranial sutures as intramembranous bone growth sites. Dev Dyn 219: 472–485. 11084647 10.1002/1097-0177(2000)9999:9999<::AID-DVDY1073>3.0.CO;2-F

[3] Sahar DE, Longaker MT, Quarto N (2005) Sox9 neural crest determinant gene controls patterning and closure of the posterior frontal cranial suture. Dev Biol 280: 344–361. 15882577 10.1016/j.ydbio.2005.01.022

[4] Moss ML (1958) Fusion of the frontal suture in the rat. Am J Anat 102: 141–166. 13545186 10.1002/aja.1001020107

[5] Logan CY, Nusse R (2004) The Wnt signaling pathway in development and disease. Annual review of cell and developmental biology 20: 781–810. 10.1146/annurev.cellbio.20.010403.113126 15473860

[6] Nelson WJ, Nusse R (2004) Convergence of Wnt, beta-catenin, and cadherin pathways. Science 303: 1483–1487. 15001769 10.1126/science.1094291 PMC3372896

[7] Akiyama H, Kim JE, Nakashima K, Balmes G, Iwai N, et al (2005) Osteo-chondroprogenitor cells are derived from Sox9 expressing precursors. Proc Natl Acad Sci U S A 102: 14665–14670. 16203988 10.1073/pnas.0504750102 PMC1239942

[8] Mori-Akiyama Y, Akiyama H, Rowitch DH, de Crombrugghe B (2003) Sox9 is required for determination of the chondrogenic cell lineage in the cranial neural crest. Proc Natl Acad Sci U S A 100: 9360–9365. 12878728 10.1073/pnas.1631288100 PMC170923

[9] Hartmann C, Tabin CJ (2001) Wnt-14 plays a pivotal role in inducing synovial joint formation in the developing appendicular skeleton. Cell 104: 341–351. 11239392 10.1016/s0092-8674(01)00222-7

[10] Hill TP, Spater D, Taketo MM, Birchmeier W, Hartmann C (2005) Canonical Wnt/beta-catenin signaling prevents osteoblasts from differentiating into chondrocytes. Dev Cell 8: 727–738. 15866163 10.1016/j.devcel.2005.02.013

[11] Ten Berge D, Brugmann SA, Helms JA, Nusse R (2008) Wnt and FGF signals interact to coordinate growth with cell fate specification during limb development. Development 135: 3247–3257. 18776145 10.1242/dev.023176 PMC2756806

[12] Behr B, Longaker MT, Quarto N (2010) Differential activation of canonical Wnt signaling determines cranial sutures fate: a novel mechanism for sagittal suture craniosynostosis. Dev Biol 344: 922–940. 20547147 10.1016/j.ydbio.2010.06.009

[13] Behr B, Longaker MT, Quarto N (2011) Craniosynostosis of coronal suture in twist1 mice occurs through endochondral ossification recapitulating the physiological closure of posterior frontal suture. Front Physiol 2: 37. 21811467 10.3389/fphys.2011.00037 PMC3143731

[14] Jho EH, Zhang T, Domon C, Joo CK, Freund JN, et al (2002) Wnt/beta-catenin/Tcf signaling induces the transcription of Axin2, a negative regulator of the signaling pathway. Mol Cell Biol 22: 1172–1183. 11809808 10.1128/MCB.22.4.1172-1183.2002 PMC134648

[15] Yu HM, Jerchow B, Sheu TJ, Liu B, Costantini F, et al (2005) The role of Axin2 in calvarial morphogenesis and craniosynostosis. Development 132: 1995–2005. 15790973 10.1242/dev.01786 PMC1828115

[16] Quarto N, Wan DC, Longaker MT (2008) Molecular mechanisms of FGF-2 inhibitory activity in the osteogenic context of mouse adipose-derived stem cells (mASCs). Bone 42: 1040–1052. 18420480 10.1016/j.bone.2008.01.026

[17] Vu TH, Shipley JM, Bergers G, Berger JE, Helms JA, et al (1998) MMP-9/gelatinase B is a key regulator of growth plate angiogenesis and apoptosis of hypertrophic chondrocytes. Cell 93: 411–422. 9590175 10.1016/s0092-8674(00)81169-1 PMC2839071

[18] Karsenty G (2003) The complexities of skeletal biology. Nature 423: 316–318. 12748648 10.1038/nature01654

[19] Day TF, Guo X, Garrett-Beal L, Yang Y (2005) Wnt/beta-catenin signaling in mesenchymal progenitors controls osteoblast and chondrocyte differentiation during vertebrate skeletogenesis. Dev Cell 8: 739–750. 15866164 10.1016/j.devcel.2005.03.016

[20] Hattori T, Muller C, Gebhard S, Bauer E, Pausch F, et al (2010) SOX9 is a major negative regulator of cartilage vascularization, bone marrow formation and endochondral ossification. Development 137: 901–911. 20179096 10.1242/dev.045203

[21] Lefebvre V, Huang W, Harley VR, Goodfellow PN, de Crombrugghe B (1997) SOX9 is a potent activator of the chondrocyte-specific enhancer of the pro alpha1(II) collagen gene. Mol Cell Biol 17: 2336–2346. 9121483 10.1128/mcb.17.4.2336 PMC232082

[22] Holmbeck K, Bianco P, Chrysovergis K, Yamada S, Birkedal-Hansen H (2003) MT1-MMP-dependent, apoptotic remodeling of unmineralized cartilage: a critical process in skeletal growth. J Cell Biol 163: 661–671. 14610065 10.1083/jcb.200307061 PMC2173657

[23] Slater BJ, Liu KJ, Kwan MD, Quarto N, Longaker MT (2009) Cranial osteogenesis and suture morphology in Xenopus laevis: a unique model system for studying craniofacial development. PLoS ONE 4: e3914. 19156194 10.1371/journal.pone.0003914 PMC2615207

[24] Jepsen KJ, Pennington DE, Lee YL, Warman M, Nadeau J (2001) Bone brittleness varies with genetic background in A/J and C57BL/6J inbred mice. J Bone Miner Res 16: 1854–1862. 11585350 10.1359/jbmr.2001.16.10.1854

